# Marine Autotroph-Herbivore Synergies: Unravelling the Roles of Macroalgae in Marine Ecosystem Dynamics

**DOI:** 10.3390/biology11081209

**Published:** 2022-08-12

**Authors:** Acga Cheng, Wai Yin Lim, Phaik-Eem Lim, Affendi Yang Amri, Sze-Wan Poong, Sze-Looi Song, Zul Ilham

**Affiliations:** 1 Institute of Biological Sciences, Faculty of Science, Universiti Malaya, Kuala Lumpur 50603, Malaysia; 2 Institute of Ocean and Earth Sciences, Universiti Malaya, Kuala Lumpur 50603, Malaysia; 3 Institute for Advanced Studies, Universiti Malaya, Kuala Lumpur 50603, Malaysia; 4 Department of Biological and Environmental Engineering, College of Agriculture and Life Sciences, Cornell University, Ithaca, NY 14850, USA

**Keywords:** autotroph-herbivore interactions, feeding behaviour, macroalgae, marine herbivores, nutrient acquisition

## Abstract

**Simple Summary:**

Invasive species are a leading hazard to marine ecosystems worldwide, coupled with climate change. Tackling the emerging biodiversity threat to maintain the ecological balance of the largest biome in the world has now become a pivotal part of the Sustainable Development Goals (SDGs). Marine herbivores are generally regarded as biological agents that restrict invasive species, and their efficiency depends on their dietary habits, especially the autotrophs they eat. Many researchers have found contradicting findings on the effects of nutritional attributes and novelty of autotrophs on herbivore eating behaviour. In light of the scattered literature on the mechanistic basis of autotroph-herbivore interactions, we provide a comprehensive review to fill knowledge gaps about synergies based on macroalgae, an important group of photosynthetic organisms in the marine biome that interact strongly with generalist herbivores. We also analyse macroalgal defence measures against herbivores, underlining unique features and potential roles in maintaining marine ecosystems. The nutritional qualities, shape, and novelty of autotrophs can alter herbivore feeding behaviour. Future research should explore aspects that can alter marine autotroph-herbivore interactions to resolve inconsistent results of specific features and the uniqueness of the organisms involved.

**Abstract:**

Species invasion is a leading threat to marine ecosystems worldwide, being deemed as one of the ultimate jeopardies for biodiversity along with climate change. Tackling the emerging biodiversity threat to maintain the ecological balance of the largest biome in the world has now become a pivotal part of the Sustainable Development Goals (SDGs). Marine herbivores are often considered as biological agents that control the spread of invasive species, and their effectiveness depends largely on factors that influence their feeding preferences, including the specific attributes of their food–the autotrophs. While the marine autotroph-herbivore interactions have been substantially discussed globally, many studies have reported contradictory findings on the effects of nutritional attributes and novelty of autotrophs on herbivore feeding behaviour. In view of the scattered literature on the mechanistic basis of autotroph-herbivore interactions, we generate a comprehensive review to furnish insights into critical knowledge gaps about the synergies based largely on the characteristics of macroalgae; an important group of photosynthetic organisms in the marine biome that interact strongly with generalist herbivores. We also discuss the key defence strategies of these macroalgae against the herbivores, highlighting their unique attributes and plausible roles in keeping the marine ecosystems intact. Overall, the feeding behaviour of herbivores can be affected by the nutritional attributes, morphology, and novelty of the autotrophs. We recommend that future research should carefully consider different factors that can potentially affect the dynamics of the marine autotroph-herbivore interactions to resolve the inconsistent results of specific attributes and novelty of the organisms involved.

## 1. Introduction

In recent decades, mounting evidence suggests that biological invasions by invasive (also called alien or non-native) species are a growing threat to global biodiversity, and is exacerbated by climate warming [1,2]. Globalization, the transformation of technological regimes and expansions of transportation networks which modify the marine habitats are other recognized drivers behind the rapid shifting of invasive species across a broad geographical range [3,4,5]. In a narrower sense, species invasions can adversely influence the dynamics of specific communities, particularly concerning the extirpation of native species [6,7] and the reduction of species richness [8]. Climate, recipient communities, and invaders are considered the prime determinants of invasion impacts, with the characteristics of recipient communities being the most critical determinant [9]. The mechanisms of invasion impact on the diversity of native species, however, are still not well understood and in fact, previous findings on invasion consequences for species richness have been contradictory; viz., either positive, negative, neutral, or multifarious impacts [9]. This invasion paradox has led to many controversial debates over the past two decades [10]. The diversity and impact of invasive species on marine ecosystems are extensively covered in a recent review by Salimi et al. (2021) [11].

Studies on aquatic ecosystems showed that the interactions between marine herbivores and various plants and/or algae (hereinafter referred to as the “autotrophs”) could reduce or even prevent the detrimental impacts of species invasions [12,13]. Lyons and Scheibling [14] reported that the establishment of the invasive green algae *Codium fragile* was enhanced by sea urchin food preference for kelps under increased water temperature and wave action, leading to increased herbivore pressure on local kelp stands. By and large, generalist marine herbivores such as most fishes and sea urchins that feed on autotrophs are common biological control agents that suppress the establishment and abundance of invasive species in the recipient communities [15,16]. It has been reported that the feeding (or grazing) preferences of the herbivores can determine the relationship between native or invasive autotrophs [17,18]. Recent findings also suggested that mechanisms underlying autotroph palatability could help resolve the inconsistent results of novelty [19,20].

Since the 1980s, efforts have been undertaken to understand the foraging behaviour of generalist marine herbivores [21,22,23,24]. Their selective foraging behaviour, which aims chiefly to regulate their nutritional needs for growth, fecundity, and performance [25,26], has been found to exert a profound impact on the biological structure of many marine ecosystems [27]. As such, theoretic insights on the nutritional relationships between herbivores and autotrophs will assist in the control and management of invasive species [28,29,30]. Generalist herbivores have also been found to make their food selection based on autotroph palatability, which depends primarily on their other unique attributes including, among others, secondary metabolites, morphology and physical stress [31,32,33,34,35]. Significant research has been devoted to examining the role and importance of some secondary metabolites in the survival and adaptation of autotrophs [36,37,38], but less attention has been paid to dissecting the value of their other attributes that may also influence the preferences of herbivores, i.e., whether to feed on native or invasive plants, or both [12]. It is worth noting that autotrophic characteristics may have the opposite effect on autotroph-herbivore interactions in controlled experimental studies where herbivores are restricted to a single autotroph species than in effects seen in field studies where herbivores are free to move around and cause natural autotroph damage. Future research examining the significance of autotroph features in interactions between autotrophs and herbivores must therefore carefully take into account the context in which the relationships have been observed [19].

In this study, relevant research papers are selected based on the pre-determined key search criteria (autotroph-herbivore interactions, feeding behaviour, macroalgae, marine herbivores, nutrient acquisition) and accessed via a reliable online database of reputable journals. One hundred and seven papers have been validated and reviewed comprehensively. At present, the available literature on the interactions between autotroph palatability and their various attributes on the nutritional ecology of marine herbivores is scattered and fragmentary [39,40,41]. It is also important to note that the chemical compounds in autotrophs that attract or deter their feeders are not well-addressed [42,43]. This review provides an overview of the unique attributes of macroalgae, which generally refers to the primary marine autotrophs and photosynthetic eukaryotes other than terrestrial plants that are capable of interacting strongly with marine herbivores [44]. Several key factors that can influence the feeding preferences of herbivores will be discussed, providing a better understanding of the interactions between autotrophs and herbivores that can potentially increase the ecological resilience of the marine biome. These are in line with the global trends in the marine sciences and sustainable development challenges, particularly in achieving the SDGs.

## 2. Marine Algae and Their Unique Attributes

**Figure 1:**
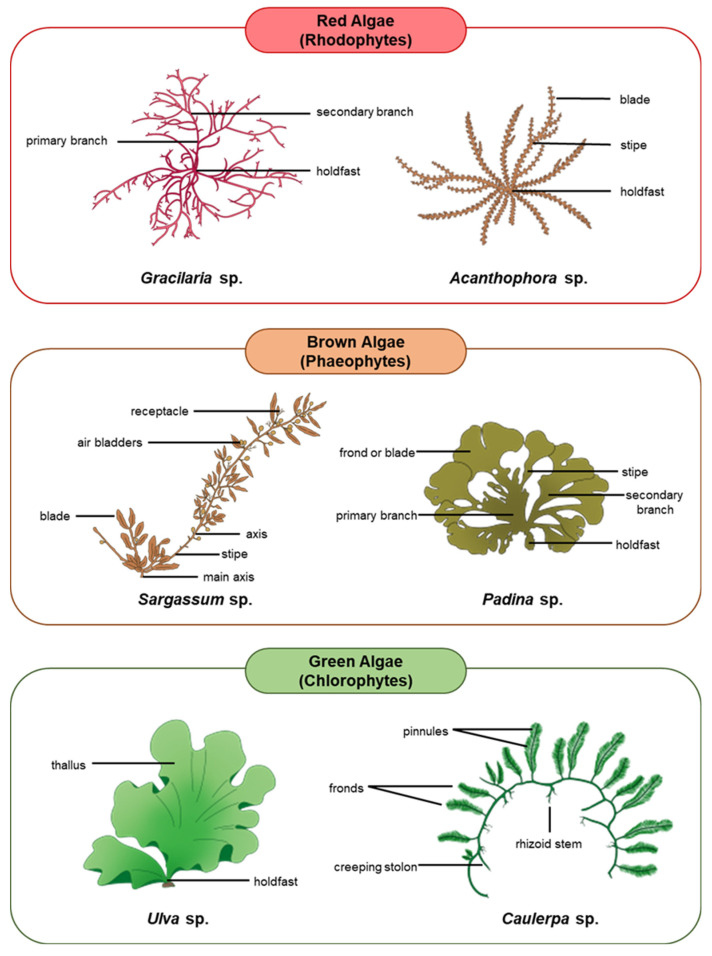
Major groups of macroalgae and examples of their common species.

Algae are the ultimate source of nutrients and energy for other organisms living in aquatic ecosystems. Although not considered plants, algae are photosynthetic in nature and produce over 70% of the global oxygen content [45,46,47]. Algae are also effective at sequestering carbon by converting almost 50% of the atmospheric carbon dioxide into organic molecules that build essential cellular constituents and intensify their energy production [48,49,50,51,52]. Macroalgae, being the most important primary producers in the oceans, house a wide range of nutritional quality within and among groups which often influences their palatability to herbivores [25,53]. For the most part, the proteins in macroalgae contain important amino acids, particularly the ones that cannot be synthesized by the animal body [54,55]. Animal hosts can thus obtain all these essential amino acids through symbiosis with the algae [56]. A variety of macroalgae reproduce either exclusively sexually or asexually, whilst some species demonstrate an alternation of generations involving both reproductive strategies in succession [57,58,59]. The following subsections discuss the unique characteristics and ecological relationships of each major group of macroalgae, including red algae (Rhodophytes), brown algae (Phaeophytes), and green algae (Chlorophytes) [51,52]. Figure 1 depicts the three major groups of macroalgae and examples of their common species.

### 2.1. Red Algae (Division Rhodophyta)

The first group is the eukaryotic red algae, or the Rhodophytes, comprising more than 6000 species of primarily marine algae ranging from microscopic to macroscopic in size [60,61]. These algae store their energy as a specialized polysaccharide, known as floridean starch, and their cell walls are made of unique cellulose and polysaccharides, such as agars and carrageenan galactans [62,63,64]. However, some other red algae may adopt sulfated mannans or neutral xylans as the main cell wall components rather than carrageenans [63]. Their photosynthetic pigments include chlorophylls *a* and *d*, while their accessory pigments are carotenoids, phycobilins, and xanthophyll [60,65,66] (Table 1). Some notable examples of red algae are, among others, filamentous species like *Pleonosporum* spp. and coralline algae like *Porolithon* spp., which contribute significantly to the building of tropical reefs and thalloid species. It is worth noting that the red algae have no flagellated cells or cells with any vestigial structure of flagellation [20]. Irish moss (*Chondrus crispus* Stackhouse), also known as the carrageen moss, is an example of an economically important red alga which has been used to bind proteins together to stabilize and add texture to various foods and beverages like ice cream, yogurt, and deli meats [67,68]. Another economically and nutritionally important species of red algae is nori (*Porphyra umbilicalis* Kützing); a high-protein and high-fibre algae which is commonly used in Japanese cuisine as an ingredient to wrap sushi [69]. *Porphyra* was proved to have the greatest protein content (ca. 35%) among the marine macroalgae, while some members of the brown algae in the order Laminariales have the lowest content (ca. 7%) [70,71].

### 2.2. Brown Algae (Division Chromophyta)

In contrast to other algal groups, brown algae or the Phaeophytes are mostly developed from a secondary endosymbiosis event which involved a non-photosynthetic eukaryote and a unicellular red alga. Resultantly, brown algae exhibit several morphological and metabolic features that make them the most complex macroalgae [72]. Phaeophytes are mostly macroscopic in size, inclusive of the giant kelp (*Macrocystis pyrifera* (Linnaeus) C.Agardh), which can grow up to 10 m in length [73]. Most of the approximately 1800 species of brown algae live in the marine environment, especially in cool temperate waters located in both the Northern and Southern Hemispheres [74,75]. Fucans and alginates are the specific polysaccharides compounds, which can be found in the cell wall of brown algae [72]. Generally, brown algae consist of three distinctly recognizable parts–the holdfast, stipe, and leaf-like blades [76]. The holdfast is a root-like structure at the bottom, which is often joined by a stipe to one or more leaf-like blades depending on the species. The blades serve as the primary surface for important processes including photosynthesis and nutrient exchange in the algae [77,78]. Although photosynthesis takes place predominantly in the blades, it is crucial that the stipe has the adequate length to place the blades sufficiently close to the light source. Alternatively, algae can absorb sufficient light by swelling the body (thallus) or increasing their growth rate [79]. The photosynthetic pigments in brown algae are chlorophylls *a* and *c*, and their accessory pigments include carotenoids and xanthophylls [80] (Table 1). Fucoxanthin contains brown-coloured pigment and the unique xanthophyll in brown algae which gives them their characteristic dark colour [81]. Unlike red algae, most of the brown algae have two flagella which help them achieve locomotion [82]. Some examples of brown algae include the rockweeds (*Ascophyllum* spp. and *Fucus* spp.) and the giant kelps (*Macrocystis* sp.). These algae usually contain laminarin and mannitol, storage sugars which can be fermented to make alcohol [83]. Some brown algae possess the ability to take up certain important substances from seawater. For instance, the iodine concentration in an edible kelp, kombu, can be thousands of times as great in the cells of the species as in its surrounding water [84].

### 2.3. Green Algae (Division Chlorophyta)

On the other hand, green algae or the Chlorophytes are generally more closely related to the higher plants in comparison to brown and red algae, in particular their chloroplast structure [85,86]. The cell walls of most species of green algae are built mainly by cellulose, with some incorporation of glycans (hemicelluloses) [87]. Their photosynthetic pigments in the chloroplast are chlorophylls *a* and *b*, while their accessory pigments are carotenoids and xanthophylls, found in embryophytes [87] (Table 1). Green algae comprise of 9000 to 12,000 species, with the majority of them occurring in freshwater rather than the marine environments [86,87]. Most green algae are microscopic, except for a small number of species in some specific genera such as those in *Cladophora* which are multicellular and macroscopic [87,88,89]. The unicellular genera *Chlamydomonas* and *Chlorella* are some common examples of green algae in both marine and freshwater ecosystems worldwide, which consist of species that disperse in a wide range of habitats [90]. An example of more complex green algae includes *Volvox*, which forms large hollow-spherical colonies that consist of thousands of cells [91]. The green algae *Ulva* spp., *Caulerpa* spp., *Enteromorpha* spp., and *Codium* spp. are commonly used as a food source for humans. The *Ulva* spp., known generally as sea lettuce, are extensively consumed in many Asian countries especially in Japan, China, and the Republic of Korea [86,92]. Access to nitrogen is one of the major limiting factors in the growth of green algae on the grounds that most of them thrive in shallow water [93]. Nevertheless, the increased runoff of fertilizer-related nitrogen into the oceans, mainly from agriculture has created favourable conditions for the growth of green algae and also other groups of algae in the past few decades [94]. According to Lee (2018), the majority of green algae form zoogametes, which are motile flagellated gametes [20]. The review by Moreira et al. (2021) details how macroalgae from various divisions differ in their flagellal construction, orientation, and life cycle in general [89].

## 3. At a Glance: Key Defence Strategies of Marine Macroalgae against Herbivores

**Figure 2:**
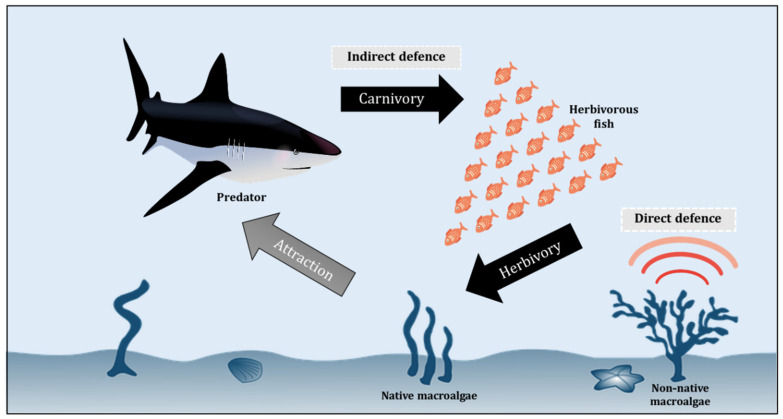
Conceptual model of macroalgae-herbivore-predator interaction.

The base of a marine food web is dominated by photosynthetic autotrophs, notably macroalgae and microalgae (phytoplankton), which are the main producers of half of the Earth’s oxygen and also the organic carbon required by all marine animals to survive [95,96,97,98]. The next level of the marine food web is made up of herbivores, from small zooplankton to larger animals (such as herbivorous fishes and manatees) that eat up a huge number of macroalgae [99,100,101]. Figure 2 illustrates a simplified conceptual model of the interaction between different levels of the marine food web, including macroalgae, herbivore, and predator, with the non-native autotroph having direct defences against herbivores. The interactions between herbivores and macroalgae are indeed one of the key drivers of marine ecosystem dynamics, gaining increasing scientific attention in recent decades (Table 2). Unfortunately, the synergies are currently being altered by climate change, affecting macroalgae growth rates and phenology, expression of chemical defenses, and herbivore behaviour and metabolism [102,103,104,105,106,107,108,109,110,111]. These macroalgae, however, have developed a variety of defence mechanisms to help them avoid herbivory and ensure their survival and abundance, as discussed in Section 3.1 and Section 3.2.

### 3.1. Physical Defences

A multitude of scientific research indicated that ocean warming has caused ecological impacts on various marine flora and fauna species across the globe, with a range of species marching away from their native homes in search of cooler climes [120,121,122,123,124,125,126,127,128,129,130,131,132,133]. With heatwaves sweeping through oceans twice as much as they did in the early 1990s, many biodiversity hotspots around the world are on the verge of imminent collapse. Many marine algae exhibit morphological plasticity that allows them to thrive in diverse habitats with various environmental pressures [134]. The study conducted by Diaz-Pulido et al. [134] showed that the morphology of different species of brown algae (*Padina boergesenii*) was significantly affected not only by herbivory but also by climatic and oceanographic factors, and this suggested that algal response to herbivory could also be a seasonal process [135,136,137,138,139]. Populations from more variable environments are considered to be more plastic [140], and algal phenotypic plasticity is potentially another pivotal mechanism that enables algae to respond to either fluctuating environments [141] or species invasion [142]. According to Fordyce [143], ecological interactions mediated by phenotypic plasticity, which are typical in nature, depend heavily on the morphological responses of the interacting organisms.

Hard encrusting calcified algae are common in the tropics where grazing is severe. Calcification of the coralline algal thallus is thought to have evolved as an adaptation to protect reproductive structures from herbivory by developing a multi-layered thallus in which reproductive structures are sunken beneath the calcareous surface cells and are thus protected from grazer access [144]. The calcified thalli may also decrease digestibility and in herbivorous herbivores (such as crabs) cause wear of chelipeds, mandibles, and the teeth of the gastric mill [145]. Increasing anthropogenic CO_2_ emissions have led to elevated oceanic pCO_2_ which may impact the structural integrity and protective function of the calcified thallus by decreasing calcification rates, and thus increasing the vulnerability of the coralline algae to bioerosion and grazing by excavating herbivores such as sea urchins and parrotfishes [146]. Non-calcifying macroalgae, on the other hand, typically use thallus toughness or mechanical strength as means of physical defence [136]. It is important to note that herbivore foraging is not essentially detrimental to the marine autotrophs. For example, limpets and chitons reportedly encourage coralline growth by regularly removing algal epiphytes from the surface of the coralline algae, which is necessary to avoid eventual overgrowing and killing of the coralline crust [147].

### 3.2. Chemical Defences

The chemical strategies of defence against herbivore are complex and generally assigned to two defence mechanisms–the direct and indirect defences [103,109]. In response to herbivory, direct defences are biologically mediated by autotroph chemistry and thus these defences can change the biological functions of the herbivores, including their feeding patterns, growth, and survival. In contrast, indirect defences against herbivory depend upon other species such as the natural enemies of the herbivores [110,111] (Figure 2). The chemical ecology of macroalgae has been widely elucidated in various regions and habitats, focusing primarily on herbivore offence and oxidative burst responses, which are chemical defences activated against pathogens and biofuels (Potin, 2008) [121,122,123]. An enormous diversity of secondary metabolites is regularly produced by autotrophs in response to herbivory in aquatic ecosystems [124,125]. Marine algae are known to be a viable source of specialized metabolites that play a crucial role in the ecosystem and climate functioning [126,127]. Tropical macroalgal taxa have been reported to produce a higher diversity of metabolites compared to their temperate counterparts, dominated by halogenated metabolites, terpenoids, acetogenins, and phenolics [127,128]. Mainly regulated by developmental, genetic, and environmental factors, these metabolites play diverse ecological functions in macroalgae, from being deterrent against herbivores to defenders to fight against specific pathogens and competitors for space with other marine organisms [127,129,130].

Over the past decade, considerable attention has been devoted to understanding the interactions between algal halogenated compound production and the environment, which includes global and anthropogenic climate changes [124,131]. Given that macroalgae produce a range of halogenated secondary metabolites, particularly chlorinated and brominated compounds that are predominant in red (90%) and green (7%) macroalgae, many studies have been conducted using these macroalgae to aid biosorption of pollutants in both industry and agriculture [124,132]. It is worth noting that halogenation of macroalgal components is involved in chemical defence mechanisms because halogenated metabolites are often associated with antibacterial, antifungal, antibacterial, and antioxidant properties [132,133].

## 4. Does Nutrient Acquisition in Algae Determine the Feeding Preferences of Marine Herbivores?

Palatability can be broadly defined as the characteristics and conditions of autotrophs that stimulate the animal to feed on them [33]. These include their structure, physical, and chemical attributes [148]. It has been long recognized that macronutrient composition influences palatability and foods that are higher in fat and protein content usually have higher palatability in terrestrial and aquatic ecosystems [149,150]. In marine communities, the preference and performance of the herbivores often relate directly to the nutritional value of algae or some other autotrophs, which is driven mostly by the protein and nitrogen content [151,152,153,154,155]. For example, several studies on the high-value marine abalone (Haliotis asinine) suggested that their diverse preferences are primarily influenced by the protein and nitrogen content of macroalgae [156,157,158,159,160,161,162,163,164,165,166,167,168,169,170,171,172]. Table 3 shows some examples of studies involving the interactions between marine autotrophs and herbivores based on the herbivore nutrient acquisition since the 2010s.

Living organisms require nitrogen to synthesize amino acids, the basic building blocks of protein that serve essential functions in virtually all biological processes [153]. Many previous studies have pointed out that low nitrogen consumption is associated with reduced food intake in generalist marine herbivores [39,148,160,161,162,163]. The study by Barile, Lapointe and Capo [156] on California sea hare (*Aplysia californica*) showed that this herbivorous gastropod preferred to feed on gracilarioid algae (*Gracilaria ferox*) with high levels of nitrogen. The lack of preference for protein-enriched algae (i.e., high-nutrient algae) can be explained by the compensatory feeding behaviour of some herbivore species. Previous studies reported the optimal growth rate and adequate intakes of limiting nutrients by testing the consumption rates of different herbivores on the low nutritional quality of algae foods [39,43,164,165]. According to Bradley et al. (2021), herbivores may avoid species that are less palatable or have lower nutritional value, which may affect their distribution and abundance [164]. However, the distinct nutritional drivers underlying the feeding preferences of specific marine herbivores are still a frontier that needs to be further explored.

## 5. Conclusions

One of the SDGs is uniquely dedicated to life below water, which is to conserve and sustainably use the oceans, seas, and marine resources for sustainable development. Nevertheless, maintaining the ecological balance of the largest biome in the world has become more challenging, especially when human-induced climate change continues to rapidly affect the diversity of marine life in an adverse way. To tackle the major threats to biodiversity, such as invasive species and habitat loss, it is worthwhile to dive into the unknown interactions between autotrophs and herbivores–those organisms that rule the base of the food chain. Theoretic insights on the synergies of autotrophs and herbivores in the marine biome are therefore crucial in controlling and managing invasive species, which almost always do more harm than good. A more concerted effort to test the major hypotheses in invasion biology, for example, the biotic resistance and enemy release hypotheses, is required to ensure the sustainability of the current marine ecosystems. Future research that aims to develop theories of marine ecology should be carefully designed, looking into various factors that can potentially affect the dynamics of different trophic levels within one or several food webs, including geographic variation and important attributes of the organisms involved. We strongly recommend the integration of evolutionary novelty theory with autotroph attributes and novelty in future studies to provide a better understanding of the consequences of biological invasions in the marine biome.

## Figures and Tables

**Table 1:** Major groups of macroalgae and their attributes.

Major Groups	Pigments	Cell Wall	Storage Components
Red algae (Rhodophytes)	Chlorophyll *a* (*d* in some Florideophyceae), R- and C- phycocyanin, allophycocyanin, R- and B-phycoerythrin, Alpha- and Beta-carotene, xanthophylls	Cellulose, xylans, galactan, alginate in corallinaceae	Floridean starch
Brown algae (Phaeophytes)	Chlorophyll *a*, *c*, Beta-carotene, fucoxanthin, xanthophylls	Cellulose, alginic acid, fucoidan	Laminaran, mannitol
Green algae (Chlorophytes)	Chlorophyll *a*, *b*, Alpha-, Beta- and Gamma- carotene, xantophylls	Cellulose, hydroxyproline glucosides, xylans, mannans, absent wall, calcified in some	Starch, oil

**Table 2:** Key findings in autotroph-herbivore studies conducted since 2000.

Location	Autotroph(s)	Herbivore(s)	Key Findings	Reference
Australia	Algal turfs	Herbivorous fishes (Acanthuridae, Scaridae and Siganidae)	Fish response mechanisms to habitat-specific differences in food production remain unclear	[112]
Caribbean and Brazil	Macroscopic algae	Herbivorous fishes (Acanthuridae and Scaridae)	Temperature-related feeding processes are most likely involved in the distribution patterns of herbivores	[113]
Caribbean-Florida	Sea grass beds	Herbivorous fishes (Acanthuridae, Scaridae, and Pomacentridae)	Robust herbivorous fish assemblages can limit reefs from further macroalgal domination	[114]
Colombia	Macroalgae	Herbivorous fishes (Gobiidae, Pomacentridae, Labridae, Mugilidae, Labrisomidae, Gobiesocidae and Muraenidae)	Small crustacean prey items dominated the diets of most species. Macroalgae and diatoms consumption by a significant number of species was also observed	[115]
Caribbean	Algal turfs	Herbivorous fishes (Acanthuridae and Scaridae)	Herbivores in promoting reef recovery and resilience may depend on their feeding preferences, abundance, and biomass	[116]
Red Sea	Macroalgae	Sea urchins and herbivorous fish	Herbivores as a crucial top-down factor in controlling both benthic algal biomass and composition	[117]
Japan	Algal beds (kelp)	Herbivorous fishes (Acanthuridae and Scaridae)	The importance of temperature-mediated fish herbivory in limiting the development of kelp populations in southern Japan is confirmed	[118]
Mediterranean Sea	Algae	Herbivorous fishes (Acanthuridae)	Expansion of tropical rabbitfishes poses a major threat to shallow water Mediterranean ecosystems	[118]
Portugal	Seagrass	Mesograzers (Amphipod and isopod)	Intraspecific variation should not be ignored when classifying a single seagrass species with respect to herbivory vulnerability. Seagrass structural traits confer mechanical resistance	[119]
Baltic Sea	Phytoplankton	Predatory zooplankton	Role of zooplankton filter feeders in controlling the development of phytoplankton	[120]
Malaysia	Macroalgae	Herbivorous fish (Chanidae)	Feeding behaviour of a herbivore could be influenced by the nutritional quality, morphology, and geography of the autotrophs	[39]

**Table 3:** Examples of studies involving marine autotroph-herbivore interactions based on herbivore nutrient acquisition since the 2010s.

Nutrient	Marine Autotroph(s)	Marine Herbivore(s)	Ref(s)
Protein	Bull kelp (*Durvillaea antarctica*)	Talitrid amphipod (*Orchestoidea tuberculate*)	[25]
	Blade tissue of bull kelp (*Durvillaea antarctica*)	Talitrid amphipod (*Orchestoidea tuberculate*)	[167]
	Red algae (*Asparagopsis taxiformis*)	Abalone (*Haliotis asinina*)	[151]
	Grey weed (*Lessonia nigrescens*)	Talitrid amphipod (*Orchestoidea tuberculate*)	[48]
	Green seaweeds	White-spotted rabbitfish (*Siganus canaliculatus*)	[168]
	Brown algae (*Sargassum* spp.)	Marine isopod (*Idotea baltica*), periwinkle (*Littorina littorea*), and green sea urchin (*Psammechinus miliaris*)	[29]
	Epiphytic red algae	Butterfish (*Odax pullus*)	[26]
	Bull kelp (*Durvillea antarctica*)	Sea snail (*Diloma nigerrima*)	[157]
Nitrogen	Sea grapes (*Caulerpa racemosa*)	Purple sea urchin (*Paracentrotus lividus*)	[169]
	Brown forkweed (*Dictyota dichotoma*)	Long-spined sea urchin (*Diadema antillarum*) and herbivorous fishes	[170]
	Brown algae (*Sargassum yezoense*)	Sea urchin (*Hemicetrotus pulcherrimus*)	[160]
	Apical portions of brown algae fronds (*Sargassum* spp.)	Parrotfish (*Sparisoma aurofrenatum* and *Sparisoma chrysopterum*)	[171]
	Green algae (*Ulva* spp.)	Purple sea urchin (*Paracentrotus lividus*)	[163]
	Marine macroalgal species near Malaysian waters	Milkfish (*Chanos chanos*)	[39]
Carbon	Sea grapes (*Caulerpa racemosa*)	Purple sea urchin (*Paracentrotus lividus*)	[169]
	Macrophyte species in Northwestern Europe	Ringed China-mark (*Parapoynx stratiotata*	[12]
	Seagrass (*Cymodocea nodosa*)	Purple sea urchin (*Paracentrotus lividus*)	[163]
Phosphorus	Macrophyte species in Northwestern Europe	Ringed China-mark (*Parapoynx stratiotata*	[12]
	Apical portions of brown algae fronds (*Sargassum* spp.)	Surgeonfish (*Acanthurus coeruleus*) and parrotfish (*Sparisoma rubripinne* and *Sparisoma chrysopterum*)	[171]
	Marine macroalgal species near Malaysian waters	Milkfish (*Chanos chanos*)	[39]
Total phenolic	Bull kelp (*Durvillaea antarctica*)	Talitrid amphipod (*Orchestoidea tuberculate*)	[25]
	Bladder wrack (*Fucus vesiculosus*)	Flat periwinkle (*Littorina obtusata*)	[172]
	Bull kelp (*Durvillaea antarctica*)	Talitrid amphipod (*Orchestoidea tuberculate*)	[25]
	Marine macroalgal species near Malaysian waters	Milkfish (*Chanos chanos*)	[39]
Secondary metabolites	Bull kelp (*Durvillaea antarctica*)	Talitrid amphipod (*Orchestoidea tuberculate*)	[25]
	Brown algae (*Sargassum yezoense*)	Sea urchin (*Hemicetrotus pulcherrimus*)	[160]
	Brown algae (*Sargassum muticum*)	Periwinkle (*Littorina littorea*), and green sea urchin (*Psammechinus miliaris*)	[29]

## Data Availability

Not applicable.
